# Developing a high-throughput phenotyping method for oxidative stress tolerance in barley roots

**DOI:** 10.1186/s13007-019-0397-9

**Published:** 2019-02-06

**Authors:** Haiyang Wang, Lana Shabala, Meixue Zhou, Sergey Shabala

**Affiliations:** Tasmanian Institute of Agriculture, University of Tasmania, Hobart, TAS 7001 Australia

**Keywords:** Salinity, Viability staining, Root growth assay, Phenotyping, Barley, Oxidative stress

## Abstract

**Background:**

More than 20% of the world’s agricultural land is affected by salinity, resulting in multibillion-dollar penalties and jeopardising food security. While the recent progress in molecular technologies has significantly advanced plant breeding for salinity stress tolerance, accurate plant phenotyping remains a bottleneck of many breeding programs. We have recently shown the existence of a strong causal link between salinity and oxidative stress tolerance in cereals (wheat and barley). Using the microelectrode ion flux estimation (MIFE) method, we have also found a major QTL conferring ROS control of ion flux in roots that coincided with the major QTL for the overall salinity stress tolerance. These findings open new (previously unexplored) prospects of improving salinity tolerance by pyramiding this trait alongside with other (traditional) mechanisms.

**Results:**

In this work, two high-throughput phenotyping methods—viability assay and root growth assay—were tested and assessed as a viable alternative to the (technically complicated) MIFE method using barley as a check species. In viability staining experiments, a dose-dependent H_2_O_2_-triggered loss of root cell viability was observed, with salt sensitive varieties showing significantly more damage to root cells. In the root growth assays, relative root length (RRL) was measured in plants grown in paper rolls under different H_2_O_2_ concentrations. The biggest difference in RRL between contrasting varieties was observed for 1 mM H_2_O_2_ treatment. Under these conditions, a significant negative correlation in the reduction in RRL and the overall salinity tolerance is reported.

**Conclusions:**

These findings offer plant breeders a convenient high throughput method to screen germplasm for oxidative stress tolerance, targeting root-based genes regulating ion homeostasis and thus conferring salinity stress tolerance in barley (and potentially other species).

**Electronic supplementary material:**

The online version of this article (10.1186/s13007-019-0397-9) contains supplementary material, which is available to authorized users.

## Background

Both global climate change and unsustainable agricultural practices resulted in significant soil salinization thus reducing crop yields [1, 2]. Until now, more than 20% of the world’s agricultural land (which accounts for 6% of the world’s total land), has been affected by excessive salts; this number is increasing daily [2, 3]. Given the fact that more food need to be acquired from the limited arable land to feed the expanding world population in the next few decades [4–6], generating crop germplasm which can grow in high-salt-content soil is considering a major avenue to fully utilise salt-affected land [7].

One of constraints imposed by salinity stress on plants is an excessive production and accumulation of reactive oxygen species (ROS), causing oxidative stress. This results in a major perturbation to cellular ionic homeostasis [8] and, in extreme cases, to a severe damage to plant lipids, DNA, proteins, pigments and enzymes [9, 10]. Plants deal with excessive ROS production by increased activity of antioxidants (AO). However, given the fact that AO profiles show strong time- and tissue- (and even organelle-specific) dependence and in 50% cases do not correlate with salinity stress tolerance [11], the use of AO activity as a biochemical marker for salt tolerance is highly questionable [12].

Recently we have shown that roots of salt-tolerant barley and wheat varieties possessed greater K^+^ retention and lower Ca^2+^ uptake when challenged with H_2_O_2_ [13]. These ionic traits were measured by using the microelectrode ion flux estimation (MIFE) technique. We have then applied this newly developed cell-based phenotyping MIFE platform to DH (double haploid) barley lines, revealing a major QTL for the above flux traits (Wang, Zhou, Shabala, unpublished observations). These findings open exciting prospects for plant breeders to screen germplasm for oxidative stress tolerance, targeting root-based genes regulating ion homeostasis and thus conferring salinity stress tolerance. The bottleneck in application of this technique in breeding programs is a currently low throughput capacity and technical complications of using the MIFE method.

The MIFE technique works as a non-invasive tool to monitor kinetics of net ion transport (uptake or release) across cellular membranes by using ion-selective microelectrodes [14]. This is based on the measurement of electrochemical gradients near the root surface. The microelectrodes are made on daily basis by the user by filling prefabricated pulled microcapillary with a sharp tip (several microns diameter) with specific backfilling solution and appropriate liquid ionophore, specific to the measured ion. Plant roots are mounted in a horizontal position in a measuring chamber, and electrodes are positioned in a proximity of the root surface using hand-controlled micromanipulators. Electrodes are then moved in a slow square-wave 12 s cycle, measuring ion diffusion profiles [15]. Net ion fluxes are then calculated based on measured voltage gradients between two positions, close to the root surface (e.g. 20 µm), and some distance (e.g. 50 µm) away. The method is skill-demanding and requires appropriate training of the personnel. The initial setup cost is relatively high (between $60,000 and $100,000, depending on a configuration and availability of axillary equipment), and the measurement of one specimen requires 20–25 min. Accounting for the additional time required for electrodes manufacturing and calibration, one operator can process between 15 and 20 specimens per business day using developed MIFE protocols [13]. As breeders are usually interested in screening hundreds of genotypes, the MIFE method in its current form is hardly applicable for such a work.

In this work, we attempted to seek much simpler alternative phenotyping methods that can be used as a proxy to screen cereal plants for oxidative stress tolerance. To do so, we developed and compared two high-throughput assays (a viability assay and a root growth assay) for oxidative stress screening of a representative cereal crop: barley (*Hordeum vulgare*). The biological rationale behind these approaches lies in a fact that ROS-induced cytosolic K^+^ depletion triggers programmed cell death [16–18] and results in the loss of cell viability. This effect is strongest in the root apex [19] and is associated with an arrest of the root growth. Reliability and feasibility of these high-throughput assays for plant breeding for oxidative stress tolerance are discussed in this paper.

## Methods

### Plant materials and growth conditions

Eleven barley (ten *Hordeum vulgare L.* and one *H. vulgare* ssp. *Spontaneum*) genotypes were used in this study. All seeds were obtained from the Australian Winter Cereal Collection (see Table 1). Seeds were surface sterilized with tenfold-diluted commercial bleach for 12 min and then rinsed for at least 30 min with a running tap water. Sterilized seeds were germinated in Petri dishes on wet filter paper for 1 day. Uniformly germinated seeds were then chosen, placed in paper rolls [20], and grown in a basic salt medium (BSM: 0.1 mM CaCl_2_ and 0.5 mM KCl, pH 5.6) in darkness at 24 ± 1 °C for another 3 days.

**Table 1:** Barley varieties used in the study

Varieties	Damage index score
SYR 01	0.25
TX 9425	1.00
CM 72	1.20
YYXT	1.45
Numar	1.70
ZUG 293	1.70
Hu 93-045	3.25
ZUG 403	5.70
Naso Nijo	7.50
Kinu Nijo 6	8.45
Unicorn	9.45

The damage index scores represent quantified damage degree of barley under salinity stress, with scores from 0 to 10 indicating barley overall salinity tolerance from the best (0) to the worst (10)

Two different types of H_2_O_2_ treatment were used. In one experiment, H_2_O_2_ was added to the beaker containing paper roll with 1 day old germinating seeds, and the treatment lasted for 3 days. In another experiment, the treatment was applied to 3-days old seedlings and lasted for 1 days, so in both cases plant seedlings were 4-days old at the time of analysis. H_2_O_2_ was added directly to BSM solution in the beaker before placing paper rolls. Concentrations of H_2_O_2_ ranged from 0 to 10 mM. Fresh solutions were made on daily basis to compensate for a possible decrease of H_2_O_2_ activity.

### Viability assay

Viability assessment of barley root cells was performed using a double staining method that included fluorescein diacetate (FDA, Cat. No. F7378, Sigma-Aldrich) and propidium iodide (PI, Cat. No. P4864, Sigma-Aldrich) [21]. Briefly, 5 mm long seminal root segments were isolated from both a root tip and a root mature zone (20–30 mm from the root tip) from control and H_2_O_2_-treated plants. The isolated segments were stained with freshly prepared 5 µg/ml FDA for 5 min followed by 3 µg/ml PI for 10 min, and washed thoroughly with distilled water. Stained root segment was placed on a microscope slide, covered with a cover slip, and assessed immediately using a fluorescent microscope. Staining and slide preparation were done in darkness. A fluorescent microscope (Leica MZ12; Leica Microsystems, Wetzlar, Germany) with I3-wavelength filter (Leica Microsystems) and illuminated by an ultra-high-pressure mercury lamp (Leica HBO Hg 100 W; Leica Microsystems) was used to examine stained root segments. The excitation and emission wavelengths for FDA and PI were 450–495 nm and 495–570 nm respectively. Photographs were taken by a digital camera (Leica DFC295, Leica Microsystems). Images were acquired and processed by LAS V3.8 software (Leica Microsystems). The exposure features of the camera were set to constant values (gain 1.0×, saturation 1.0, gamma 1.0) in each experiment, allowing direct comparison of various genotypes. For untreated roots, the exposure time was 591 ms; for H_2_O_2_-treated roots it was increased to 1.9 s. The overview of the experimental protocol for viability assay by the FDA - PI double staining method can be found in Additional file 1: Figure S1.

### Fluorescence intensity quantification

The *ImageJ* software was used to quantify red fluorescence intensity that is indicative of the proportion of dead cells [22]. The acquired fluorescent image was opened in *ImageJ* and colour channel of the image were split into green, red and blue. The “polygon selections” from the tool bar of *ImageJ* was used to select an area from root elongation (0.3 mm from root tip) and/or mature zone (anywhere of the cut mature root segments) from the image with red channel. The measurement was conducted by going to “Analyze” option at the main menu, followed by the “Measure” option. The results will be shown in a pop-up window containing the size of the measuring area chosen, the minimum/maximum/mean value of the red fluorescence intensity. Images of H_2_O_2_-treated roots were normalised using control (untreated) roots as a background. The red fluorescence intensity (mean value) was recorded as removal of intensity in untreated roots from H_2_O_2_-treated root in arbitrary units.

### Root growth assay

The length of the longest seminal root was measured form 4-days old barley seedlings using a ruler after 3 days of treatments with various concentrations of H_2_O_2_ ranging between 0 and 10 mM (0, 0.1, 0.3, 1, 3, 10 mM). The relative root lengths (RRL) were estimated as percentage of root lengths to controls of the respective genotypes.

### The damage index score

The damage index was used as a measure of the overall salt stress tolerance. This index is based on the extent of leaf necrosis and chlorosis in plants exposed to prolonged (5–6 weeks) salinity and quantified on 0–10 scale (0—no visual stress symptoms; 10—dead plants) [23–26]. Being measured at the vegetative stage, this damage index correlates significantly with the relative grain yield at the time of harvest and thus is a convenient proxy for quantification of plant salinity stress tolerance [13, 25].

### Statistical analysis

Statistical significance of mean values was determined by the standard Student’s *t* test at *P* < 0.05 level.

## Results

### H_2_O_2_ causes loss of the cell viability in a dose-dependent manner

**Fig. 1:**
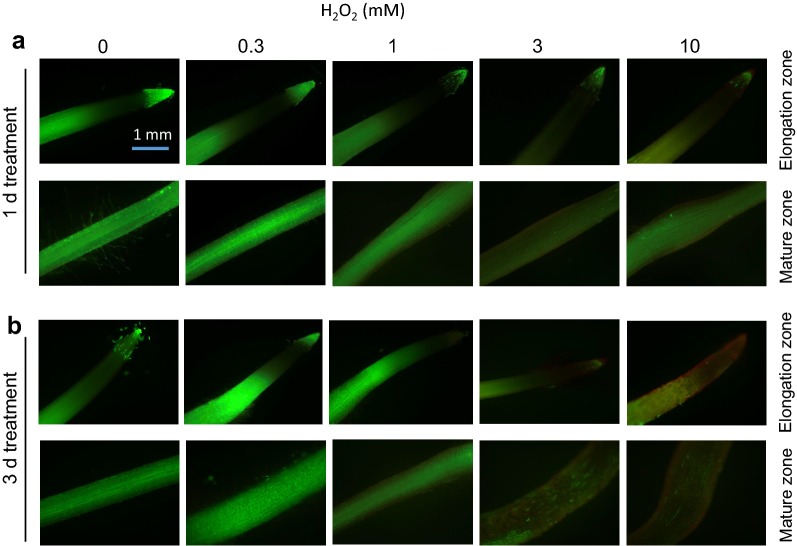
Viability staining of Naso Nijo roots (elongation and mature zones) exposed to 0, 0.3, 1, 3, 10 mM H_2_O_2_ for 1 day (**a**) and 3 days (**b**). One (of five) typical images is shown from each concentration and root zone. Bar = 1 mm

Barley variety Naso Nijo was used to study dose-dependent effects of H_2_O_2_ on cell viability. The concentrations of H_2_O_2_ used was from 0.3 to 10 mM. Both 1 day- (Fig. 1a) and 3 days- (Fig. 1b) exposure to oxidative stress caused dose-dependent loss of the root cell viability. One-day H_2_O_2_ treatment was less severe and was observed only at the highest H_2_O_2_ concentration used (Fig. 1a). When roots were treated with H_2_O_2_ for 3 days, the red fluorescence signal can be readily observed from H_2_O_2_ treatments above 3 mM (Fig. 1b).

**Fig. 2:**
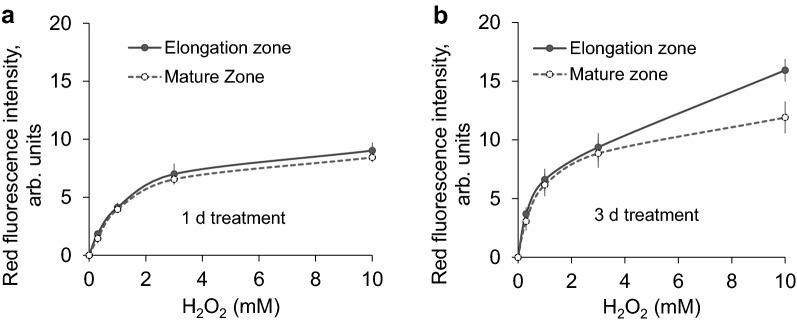
Red fluorescence intensity (in arbitrary units) measured from roots of Naso Nijo upon exposure to various H_2_O_2_ concentrations for either 1 day (**a**) or 3 days (**b**). Mean ± SE (n = 5 individual plants)

The quantitative analysis of the red fluorescence intensity was implemented in order to translate images into numerical values (Fig. 2). Mild root damage was observed upon 1 day H_2_O_2_ treatment, and there was no significant difference between elongation zone and mature zone for any concentration used (Fig. 2a). Similar findings (e.g. no difference between two zones) were observed in 3 days H_2_O_2_ treatment when the concentration was low (≤ 3 mM) (Fig. 2b). Application of 10 mM H_2_O_2_ resulted in severe damage to root cells and clearly differentiated the insensitivity difference between the two root zones, with elongation zone showing more severe root damage compared to the mature zone (Fig. 2b; significant at *P* < 0.05). Accordingly, 10 mM H_2_O_2_ with 3 days treatment was chosen as the optimum experimental treatment for viability staining assays on contrasting barley varieties.

### Genetic variability of root cell viability in response to 10 mM H_2_O_2_

**Fig. 3:**
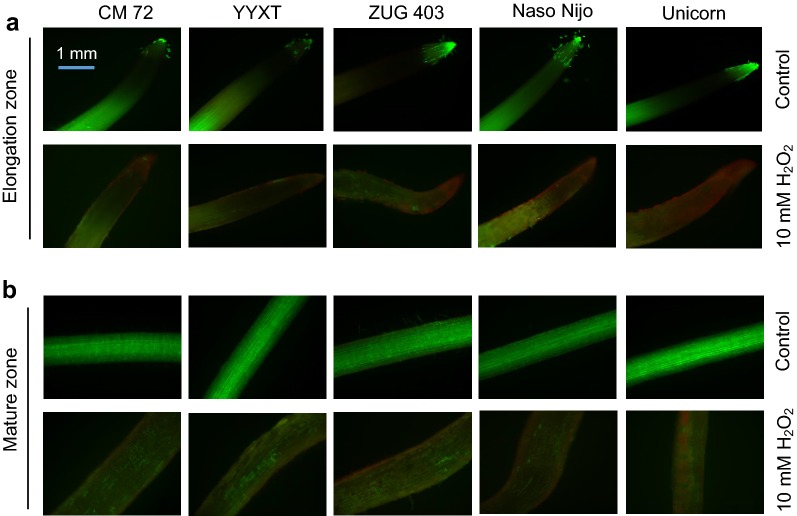
Viability staining of root elongation (**a**) and mature (**b**) zones of four barley varieties (CM 72, YYXT, ZUG 403, Unicorn) exposed to 10 mM H_2_O_2_ for 3 days. One (of five) typical images is shown for each zone. Bar = 1 mm

Five contrasting barley varieties (salt tolerant: CM 72 and YYXT; salt sensitive: ZUG 403, Naso Nijo and Unicorn) were employed to explore the extent of root damage upon oxidative stress by the means of viability staining of both elongation and mature root zones. A visual assessment showed a clear root damage upon 3 days-exposure to 10 mM H_2_O_2_ in all barley varieties and both root zones, and damage in the elongation zone was more severe than in the mature zone (Fig. 3).

**Fig. 4:**
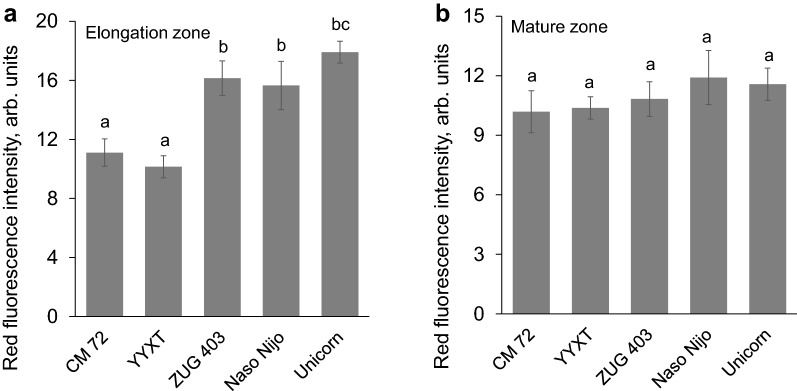
Quantitative red fluorescence intensity from root elongation (**a**) and mature zones (**b**) of five barley varieties exposed to 10 mM H_2_O_2_ for 3 days. Mean ± SE (*n* = 6)

The quantitative analysis of the fluorescence intensity revealed that salt sensitive varieties showed stronger red fluorescence signal in the root elongation zone than tolerant ones (Fig. 4a), indicating much severe root damage in the former genotypes. However, root damage between CM 72 and YYXT was insignificant. The similar trend was also found among salt sensitive varieties ZUG 403, Naso Nijo and Unicorn (Fig. 4a). In mature root zone, no significant difference was observed amongst the root cell viability of five contrasting varieties studied (Fig. 4b).

The results in this section were consistent with our previous findings by using MIFE technique [13], which elucidated that not only oxidative stress-induced transient ions fluxes but also long-term root damage may correlate with the overall salinity tolerance in barley. Before this conclusion is generalized and make it applicable to other plant species, it requires further validation using larger number of plant accessions. However, given the fact that the magnitude of H_2_O_2_-induced K^+^ and Ca^2+^ fluxes were about the same for barley and wheat [13], we have all the reasons to believe the required modifications will be rather minor.

Based on these findings, we can conclude that plant oxidative and salinity stress tolerance in barley may be quantified by the viability staining of roots treated with 10 mM H_2_O_2_ for 3 days that would include staining the root tips with FDA and PI, and then quantifying intensity of the red fluorescence signal (dead cells) from the root elongation zone. This protocol is simpler and quicker than MIFE assessment and requires only a few minutes of measurements per sample, making this assay compliant with the requirements for high throughput assays.

### Methodological experiments for cereal screening in root growth upon oxidative stress

**Fig. 5:**
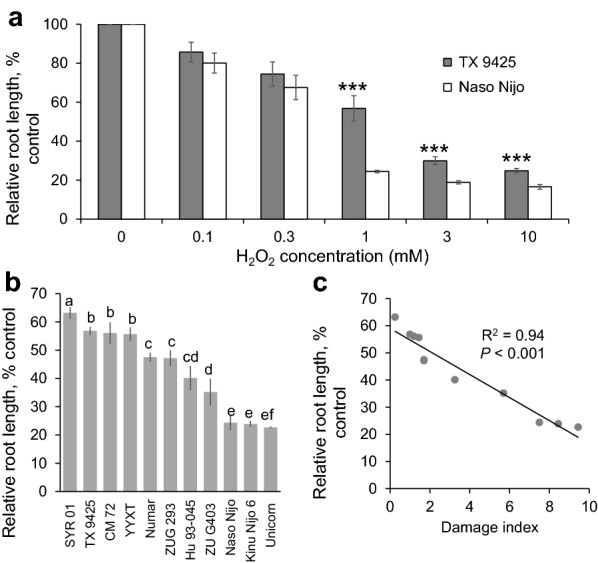
**a** Relative root length of TX 9425 and Naso Nijo seedlings treated with 0, 0.1, 0.3, 1, 3, 10 mM H_2_O_2_ for 3 days. Mean ± SE (*n* = 20). Asterisks indicate statistically significant differences between two varieties at *P* < 0.001 (Student’s *t* test). **b** Genetic variability in the relative root length in 11 barley varieties treated with 1 mM H_2_O_2_ for 3 days. Mean ± SE (*n* = 20). **c** Correlation between H_2_O_2_–treated relative root length and the overall salinity tolerance (damage index, see Table 1) of 11 barley varieties

Being a high throughput in nature, the above imaging assay still requires sophisticated and costly equipment (e.g. high-quality fluorescence camera; microscope etc.), and thus may be not easily applicable by all the breeders. This has prompted us to go along another avenue by testing root growth assays. Two contrasting barley varieties, TX 9425 (salt tolerant) and Naso Nijo (salt sensitive) were used for standardizing concentration of ROS (H_2_O_2_) treatment in preliminary experiments. After 3 days of H_2_O_2_ treatment, root length declined in both the varieties for any given concentration tested (0.1, 0.3, 1, 3, 10 mM), and salt tolerant variety TX 9425 grew better (had higher relative root length, RRL) than salt sensitive variety Naso Nijo at each treatment used (Fig. 5a). The decreased RRL showed the dose-dependency upon increasing H_2_O_2_ concentration, with a strong difference (*P* < 0.001) occurring from 1 to 10 mM H_2_O_2_ treatments between the contrasting varieties (Fig. 5a). The biggest difference in RRL between the varieties was observed under 1 mM H_2_O_2_ treatment (Fig. 5a), which was chosen for screening assays.

### H_2_O_2_-induced changes of root length correlate with the overall salinity tolerance

Eleven barley varieties were selected to test the relationship between the root growth under oxidative stress and their overall salinity tolerance under 1 mM H_2_O_2_ treatment. After 3 days exposure to 1 mM H_2_O_2_, the relative root length (RRL) of all the barley varieties reduced rapidly, ranging from the lowest 22.7% ± 0.3 (in the variety Unicorn) to the highest 63.2% ± 2 (in SYR01) (Fig. 5b). The RRL values were then correlated with the “damage index” scores (Table 1). A significant correlation (*r*^*2*^ = 0.94, *P* < 0.001) between RRL and the overall salinity tolerance was observed (Fig. 5c), indicating a strong suitability of the RRL assay method as a proxy for oxidative/salinity stress tolerance. Given the “no cost no skill” nature of this method, it can be easily taken on board by plant breeders for screening the germplasm and mapping QTLs for oxidative stress tolerance (one of components of the salt tolerance mechanism).

## Discussion

### H_2_O_2_ causes a loss of the cell viability and decline of growth in barley roots

H_2_O_2_ is one of the major ROS produced in plant tissues under stress conditions that leads to oxidative damage. The effect of this stable oxidant on plant cell viability and root growth was investigated in this study. Both parameters decreased in a dose- and/or time-dependent manner upon H_2_O_2_ exposure (Figs. 1, 5a). The physiological rationale behind these observations may lay in a fact that exogenous application of H_2_O_2_ causes instantaneous [K^+^]_cyt_ and [Ca^2+^]_cyt_ changes in different root zones [13].

Stress-induced enhanced K^+^ leakage from root epidermis results in depletion of cytosolic K^+^ pool [27] thus activating caspase-like proteases and endonucleases and triggering PCD [17, 28], leading to deleterious effect on plant viability [29]. This is reflected in our findings that roots lost their viability after being treated with H_2_O_2_, especially upon higher dosage and long-term exposure (Fig. 2). Furthermore, K^+^ is required for root cell expansion [30] and plays a key role in stimulating growth [31, 32]. Therefore, the loss of a large quantity of cytosolic K^+^ might be the primary reason for the inhibition of the root elongation in our experiments (Fig. 5a, b). This is consistent with root growth retardation observed in plants grown in low-K^+^ media [33].

High concentration of cytosolic K^+^ is essential for optimizing plant growth and development. Also essential is maintenance of stable (and relatively low) levels of cytosolic free Ca^2+^ [34, 35]. Therefore, H_2_O_2_-induced cytosolic Ca^2+^ disequilibrium may be another contributing factor to the observed loss of cell viability and reported decrease in the relative root length in this study (Figs. 3, 5a, b). Previously we showed that plants responded to H_2_O_2_ by increased Ca^2+^ uptake in mature root epidermis [13]. This is expected to result in [Ca^2+^]_cyt_ elevation that may be deleterious to plants, as it causes protein and nucleic acids aggregation, initiates phosphates precipitation and affects the integrity of the lipid membranes [36]. It may also make cell walls less plastic through rigidification, thus inhibiting cell growth [34]. In root tips, however, increased Ca^2+^ loading is required for the stimulation of actin/myosin interaction to accelerate exocytosis that sustains cell expansion and elongation [37]. The *rhd2* Arabidopsis mutant lacking functional NADPH oxidase exhibited stunted roots as plants were unable to produce sufficient ROS to activate Ca^2+^-permeable NSCCs to enable Ca^2+^ loading into the cytosol [38].

### Salt tolerant barley roots possess higher root viability in the elongation zone after long-term ROS exposure

It was argued that the ROS-induced self-amplification mechanism between Ca^2+^-activated NADPH oxidases and ROS-activated Ca^2+^-permeable cation channels in the plasma membrane, and transient K^+^ leakage from cytosol, may be both essential for the early stress signalling [29, 39, 40]. As salt sensing mechansim is most likely located in the root meristem [41], this may explain why the correlation between the overall salinity tolerance and H_2_O_2_-induced transient ions fluxes was not found in this zone in short-term experiments [13]. Under long-term H_2_O_2_ exposures, however (as in this study), we observed less severe root damage in the elongation zone in salt tolerant varieties (Fig. 4a). This suggested a possible recovery of these genotypes from the “hibernated state” (transferred from normal metabolism by reducing cytosolic K^+^ and Ca^2+^ content for salt stress acclimation) to stress defence mechanisms [42], which may include a superior capability in maintaining more negative membrane potential and increasing the production of metabolites in this zone [19]. This is consistent with a notion of salt tolerant genotypes being capable of maintaining more negative membrane potential values resulting from higher H^+^-ATPases activity in many species [43–45], and the fact that a QTL for the membrane potential in root epidermal cells was co-located with a major QTL for the overall salinity stress tolerance [46].

In the mature root zone, the salt-sensitive varieties possessed a higher transient K^+^ efflux in response to H_2_O_2_ [13]; yet no major difference in viability staining was observed amongst the genotypes in this root zone after a long-term (3 days) H_2_O_2_ exposure (Figs. 3b, 4b). This is counterintuitive and suggests an involvement of some additional mechanisms. One of these mechanisms may be a replenishing of the cytosolic K^+^ pool on the expense of the vacuole. As a major ionic osmoticum in both the cytosolic and vacuolar pools, potassium has a significant role in maintaining cell turgor, especially in the latter compartment [47]. Increasing cytosolic Ca^2+^ was first shown to activate voltage-independent vacuolar K^+^-selective (VK) channels in *Vicia Faba* guard cells [48], mediating K^+^ back leak into cytosol from the vacuole pool. This observation was later extended to cell types isolated from Arabidopsis shoot and root tissues [49], as well as other species such as barley, rice and tobacco [50]. Thus, the higher Ca^2+^ influx in sensitive varieties upon H_2_O_2_ treatment is expected to increase their cytosolic free Ca^2+^ concentration thus inducing a strong K^+^ leak from the vacuole, to compensate for the cytosolic K^+^ loss from ROS-activated GORK channel. This process will be attenuated in the salt tolerant varieties, that have lower H_2_O_2_-induced Ca^2+^ uptake. As a result, 3 days after the stress onset, the amount of K^+^ in the cytosol in mature root zone may be not different between contrasting varieties, explaining the lack of difference in viability staining.

### Evaluating root growth assay screening for oxidative stress tolerance

A rapid and revolutionary progress in plant molecular breeding has been witnessed since the development of molecular markers in the 1980s [51]. At the same time, the progress in plant phenotyping has been much slower and in most cases lack direct causal relationship with the traits targeted. However, future breeding programmes are in a need of a sensitive, low cost and efficient high-throughput phenotyping methods. The novel approach developed in our previous study allowed to use the MIFE technique for the cell-based phenotyping [13] for root sensitivity to ROS, one of the key components of mechanism of salinity stress tolerance. Being extremely sensitive and allowing to directly target operation of specific transport proteins, this method is highly sophisticated and is not expected to be easily embraced by breeders. In this study, we provided an alternative approach, namely root growth assay, which can be used as the high-throughput phenotyping method to replace the sophisticated MIFE technique. This screening method has minimal space requirements (only a small growth room) and no measuring equipment except a simple ruler. Assuming one can acquire five length measurements per minute and 20 biological replicates are sufficient for one genotype, the time needed for one genotype is just four minutes, which means one can finish the screening of 100 varieties within one working day. This is a blazing fast avenue compared to most other methods. This offers plant breeders a convenient assay to screen germplasm for oxidative stress tolerance and identify root-based QTLs regulating ion homeostasis and conferring salinity stress tolerance.

In this work, this method was only tested in barley used as a check species. However, given the fact that salt-induced increase in ROS production in roots is a general biological phenomenon [52–54], we expect that results of this study may be extrapolated to other plant species including all major cereal crops, after fine-tuning of the exposure times and concentrations of H_2_O_2_ used and acquiring the damage index scores of these plants.

## Conclusions

Our results show that oxidative and salinity stress tolerance phenotyping in barley may be conducted by staining with FDA and PI and then quantifying the intensity of red fluorescence signal from H_2_O_2_-treated root elongation zone, or by measuring roots lengths after the same treatment with the former. However, the latter method is low/no-cost and easy-to-implement thus may be recommended as an alternative phenotyping method in future screening programs.

## Additional file

**Additional file 1: Fig. S1.** Flowchart of viability staining and fluorescence image acquisition. **a** Preparation of root sample for viability staining. (From left to right panel) Surfaced sterilized seeds were germinated in a large Petri dish with wet filter paper for 1 day. Uniformly germinated seeds were then chosen and placed in paper rolls before placing it in a beaker with growth media for another 3 days. Isolated root segments were placed in a micro Petri dish containing 5 µg/ml FDA for 5 min and then transferred to another micro Petri dish containing 3 µg/ml PI for 10 min; Stained root segments were washed with distilled water and positioned on a glass slide and covered with a cover slip. **b** The prepared slide was placed on a fluorescent microscope mechanical stage under the fluorescent light and root fluorescent image was acquired by the LAS V3.8 software.

## Abbreviations

MIFE: microelectrode ion flux estimation
QTL: quantitative trait locus
ROS: reactive oxygen species
RRL: relative root length
AO: antioxidants
DH: double haploid
FDA: fluorescein diacetate
PI: propidium iodide
NSCCs: non-selective cation channels
PCD: programmed cell death
VK: vacuolar K^+^-selective channels
GORK: guard cell outward rectifying K^+^ channel
