# Supplementary figures and images for: Developing a high-throughput phenotyping method for oxidative stress tolerance in barley roots
Source: Plant Methods. 2019 Feb 6;15:12. doi: 10.1186/s13007-019-0397-9 (PMC6364415; doi:10.1186/s13007-019-0397-9)

## Slide 1
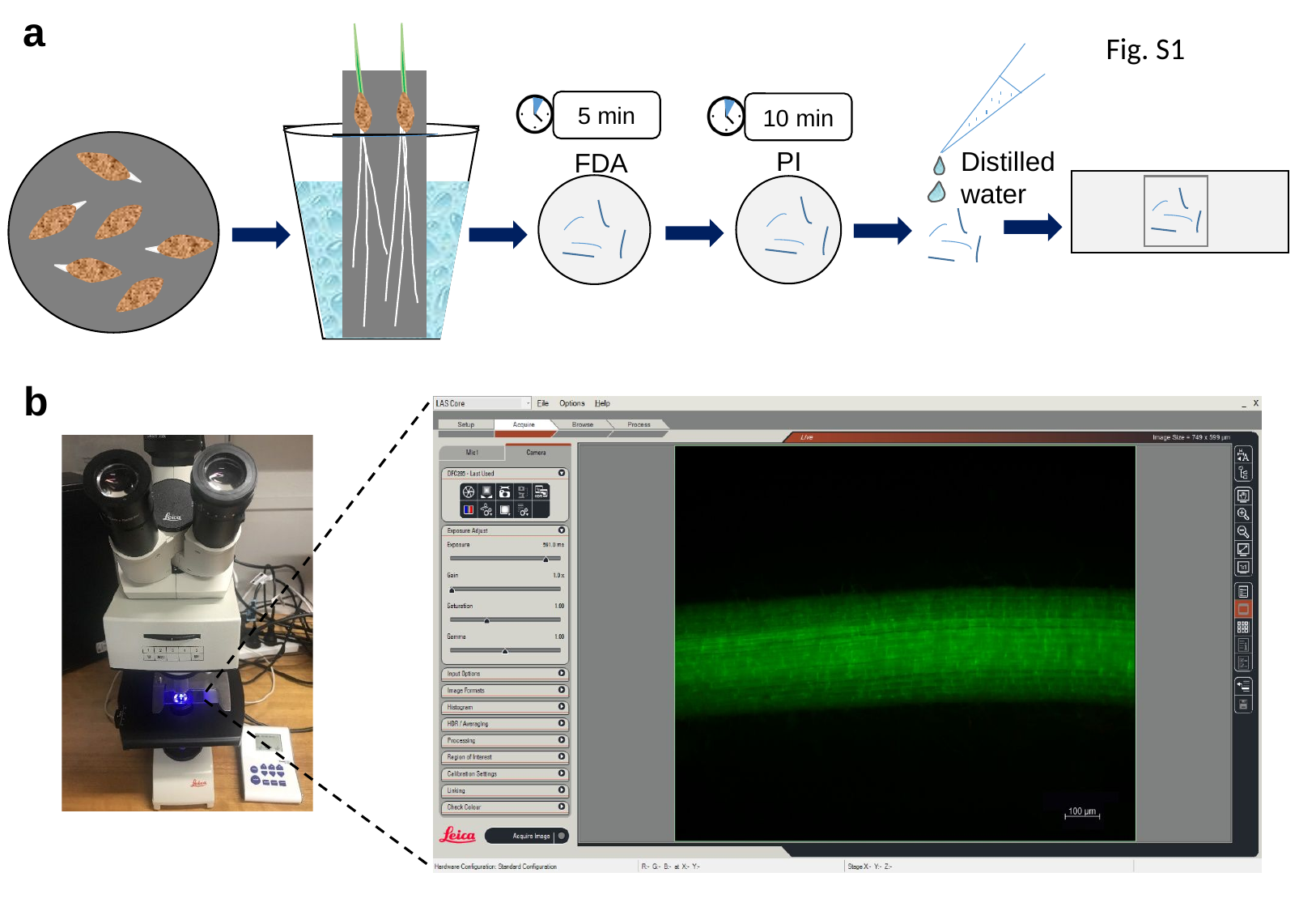

a
5 min
10 min
PI
Distilled water
FDA
b
Fig. S1

Supplement: Supplementary file 1 — Additional file 1: Fig. S1. Flowchart of viability staining and fluorescence image acquisition. a Preparation of root sample for viability staining. (From left to right panel) Surfaced sterilized seeds were germinated in a large Petri dish with wet filter paper for 1 day. Uniformly germinated seeds were then chosen and placed in paper rolls before placing it in a beaker with growth media for another 3 days. Isolated root segments were placed in a micro Petri dish containing 5 µg/ml FDA for 5 min and then transferred to another micro Petri dish containing 3 µg/ml PI for 10 min; Stained root segments were washed with distilled water and positioned on a glass slide and covered with a cover slip. b The prepared slide was placed on a fluorescent microscope mechanical stage under the fluorescent light and root fluorescent image was acquired by the LAS V3.8 software. [file 13007_2019_397_MOESM1_ESM.pptx]
